# Trend of preventable deaths up to the 6th day of life in the state of São Paulo – 2008 to 2017

**DOI:** 10.11606/s1518-8787.2020054002309

**Published:** 2020-11-27

**Authors:** Arnaldo Sala, Carla Gianna Luppi

**Affiliations:** I Secretaria de Estado da Saúde Coordenadoria de Regiões de Saúde/Atenção Básica São PauloSP Brasil Secretaria de Estado da Saúde. Coordenadoria de Regiões de Saúde/Atenção Básica. São Paulo, SP, Brasil; II Universidade Federal de São Paulo Escola Paulista de Medicina Departamento de Medicina Preventiva São PauloSP Brasil Universidade Federal de São Paulo. Escola Paulista de Medicina. Departamento de Medicina Preventiva. São Paulo, SP, Brasil

**Keywords:** Early Neonatal Mortality, Midwifery, Integrated Management of Childhood Illness, Maternal-Child Health Services, Ecological Studies

## Abstract

**OBJECTIVE:**

To analyze the trend of early neonatal infant mortality in the state of São Paulo according to preventability and region of residence.

**METHODS:**

Ecological study with secondary data from 2008 to 2017, obtained from the *Sistema de Informação sobre Nascidos Vivos* (Sinasc – Live Birth Information System) and the *Sistema de Informação sobre Mortalidade* (SIM – Mortality Information System). The causes of death were classified according to preventability groups, and the annual percentage changes in the death rates of each preventability group were estimated using the Joinpoint software.

**RESULTS:**

The early neonatal component showed a reduction trend with an annual percentage change of −1.18 (95%CI −1.63 to −0.72), less pronounced than the other age components of infant mortality. In the analysis according to preventability, the causes reducible by attention to the woman during pregnancy and those reducible by attention to the fetus and the newborn presented annual percentage change of −1.03 (95%CI: −1.92 to −0.13) and −2.6 (95%CI: −4.07 to −1.11), respectively. In the causes reducible by attention to women during delivery, no reduction trend was observed. Regional discrepancies occurred in the variation of early neonatal infant mortality rates according to type of preventability.

**CONCLUSIONS:**

Mortality up to the 6th day of life presented greater difficulty of reduction when compared with the other age components. The absence of a reduction trend in preventable deaths due to the attention to women during delivery points to possible fragility in the attention to delivery.

## INTRODUCTION

The reduction of infant mortality rates (IMR) has been the subject of concern of health managers and policy makers. Brazil has met the target set in the United Nations Millennium Development Goals for IMR ^1^ . In 2015, the 2030 Agenda for Sustainable Development ^2^ was established, which reaffirms the concern with the reduction of deaths of children under five years of age, with emphasis on neonatal mortality ^3^^,^^4^ . In this context, access to a quality health care becomes essential, pointing to the reduction of maternal, infant and neonatal deaths, mitigating preventable deaths.

In Brazil, IMR has declined in recent decades, with 29.0 deaths per 1,000 live births in 2000, reducing to 17.2 deaths per 1,000 live births in 2010 and reaching 12.8 deaths per 1,000 live births in 2017 ^5^ . The IMR in the state of São Paulo has also shown a consistent reduction over the last decades, with 16.07 deaths per 1,000 live births in 2001, reducing to 11.86 deaths per thousand live births in 2010 and reaching 10.90 deaths per 1,000 live births in 2017 ^6^ .

Neonatal and early neonatal mortality (deaths before the 7º day of life) showed significant reduction in the last decade in the state of São Paulo. However, this reduction was less pronounced between 2011 and 2017: from 7.90 to 7.66 neonatal deaths per 1,000 live births in 2017 ^6^ .

Infant deaths up to the 27th day of life corresponded to about 68% to 70% of the total infant deaths in this same period. Those that occurred until the 6th day of life accounted for 49% to 51% of the total number of infant deaths ^6^ .

In a study reviewing infant mortality in Brazil, Santos et al. ^7^ point to the change between 1980 and 1983 and in the most recent period, from 2005 to 2008, in which the factors related to the newborn, maternal, care and socioeconomic factors stand out. Although a significant proportion of the factors associated with neonatal mortality are associated with biological and sociodemographic aspects of the newborn ^8^^,^^9^ , it is important to consider the issue of the preventable nature of these deaths, with reference to the access and quality of care resources available for follow-up during pregnancy, as well as the resources available for delivery and newborn care.

Malta and Duarte ^10^ consider the classification of preventability, or preventable cause of death, when death is preventable by action of health services. In Brazil, A list of preventable causes of death due to interventions of the Unified Health System was published in 2008, which was later updated ^11^ .

Since challenges for the reduction of infant mortality and, particularly, early neonatal mortality still remain, our study aims to analyze the trends in early neonatal infant mortality rates (ENIM) according to the classification of preventable causes of death by SUS interventions for the state of São Paulo and verify the behavior of these rates in each of the administrative regions that correspond to the Regional Departments of Health (RDH) of the São Paulo State Department of Health.

## METHODS

This is an ecological time series study of infant mortality rates of the total infant deaths occurred in the state of São Paulo between 2008 and 2017.

The source of data on infant deaths was the *Sistema de Informação de Mortalidade* (SIM – mortality Information System) database available for download on the Datasusᵃ. Live births data came from the *Sistema de Informação de Nascidos Vivos* (Sinasc – Live Births Information System), consolidated on the Datasus website, in Health Information (Tabnet), Vital Statistics subsection ^12^ . The year 2017 was the most recent available on the DATASUS website; thus, since this is a time series study, the last 10 available years were considered.

The variables included in the analysis were: age of death, underlying cause of death and the individual's residence RDH. Age of death was considered to identify the three age components of infant mortality: ENIM from 0 to 6 days, late neonatal infant mortality (LNIM) from 7 to 27 days and late infant mortality (LIM) from 28 to 364 days.

The underlying cause of death codified by the International Classification of diseases (ICD) was grouped according to the Brazilian list of preventable causes of death by SUS interventions for the age group from 0 to 4 years ^11^ . This list consists of three groups of causes: Group 1 – preventable causes (divided into 6 subgroups); Group 2 – ill-defined causes of death; Group 3 – other causes (not clearly preventable).

The municipalities of residence were grouped according to the areas of coverage of the RDH that constitute the administrative structure of the São Paulo State Department of Health. The municipality of São Paulo was separated from the RDH of Greater São Paulo due to its population magnitude, resulting in 18 regions.

The death rates per 1,000 live births were estimated for each year included in our study, as well as the rates for each of the three age components. For the specific analysis of early neonatal deaths, specific death rates were estimated for each group and subgroup of preventable causes of death.

The data were initially tabulated using IBM SPSS Statistics 20 software. The mortality rates were estimated using Microsoft Excel software, version 2010.

Trends in infant mortality rates in each age component were analyzed. For ENIM, trends in rates specific to each group and subgroup of preventable causes of death were analyzed. In the analysis of ENIM in the areas of RDH, only the trends for the three subgroups of preventability with greater relevance for deaths occurring up to the 6th day of life were analyzed: reducible by adequate attention to the woman in pregnancy, reducible by adequate attention to the woman in delivery and reducible by adequate attention to the fetus and newborn.

The trends were analyzed by estimating the annual percent change (APC) of mortality rates from 2008 to 2017, using the Joinpoint Regression program 4.7.0.0 software, adopting a log-linear model with the year of death as an independent variable and the different rates as dependent variables. The software identifies any inflection points (joinpoints) on the trend curve, when significant change in the inflection of the curve occurs. In the occurrence of joinpoints, the average annual percent change (AAPC) for the period is also estimated. A 95% confidence interval (CI) were adopted.

The database used is available for public access at http://datasus.saude.gov.br/informacoes-de-saude/servicos2/transferencia-de-arquivos and it has no field with data identifying people individually. Because it is a publicly accessible database without individual identification, in accordance with Law No. 12,527, of November 18, 2011, it was not necessary to evaluate it by the research ethics committee, in accordance with Resolution No. 510/2016 of the National Health Council.

## RESULTS

In the years 2008 and 2017, 70,858 infant deaths were recorded, with 35,071 occurring until the 6th day of life, 13,767 occurring between the 7th and 27th day of life and 22,020 occurring with 28 or more days of life. The IMR ranged from 12.60 in 2008 to 10.92 deaths per 1,000 live births in 2017. ENIM participated with 48.8% of deaths in 2008 to 50.4% in 2017. ENIM rates ranged from 6.14 in 2008 to 5.51 deaths per 1,000 live births in 2017 ( Chart 1 ).

**Chart 1:** Infant mortality rates according to age components and according to groups and subgroups of preventable causes of death by interventions of the Brazilian national health system for early neonatal infant mortality, state of São Paulo, from 2008 to 2017.

Component/preventable causes of death		Year of death									
		2008	2009	2010	2011	2012	2013	2014	2015	2016	2017
Early neonatal infant mortality		6.14	6.13	5.75	5.72	5.74	5.67	5.73	5.51	5.50	5.51
	1.1. Reducible by immunoprevention actions	0.00	0.00	–	–	–	–	–	–	–	–
	1.2.1. Reducible by adequate attention to the woman during pregnancy	2.40	2.32	2.21	2.31	2.22	2.38	2.30	2.22	2.07	2.15
	1.2.2. Reducible by adequate attention to the woman during delivery	0.84	0.86	0.86	0.72	0.81	0.75	0.74	0.73	0.80	0.84
	1.2.3. Reducible by adequate attention to the fetus and the newborn	1.31	1.34	1.25	1.28	1.26	1.04	1.12	1.03	1.14	1.09
	1.3. Reducible by actions of diagnosis and adequate treatment	0.02	0.02	0.01	0.01	0.01	0.00	0.01	0.02	0.00	0.01
	1.4. Reducible by health promotion actions linked to health care actions	0.03	0.03	0.02	0.03	0.03	0.04	0.02	0.02	0.03	0.02
	2. Ill-defined causes	0.20	0.17	0.17	0.10	0.12	0.16	0.15	0.15	0.14	0.16
	3. Other causes (not clearly preventable)	1.35	1.38	1.23	1.28	1.30	1.30	1.39	1.34	1.32	1.24
Late neonatal infant mortality		2.45	2.54	2.39	2.19	2.19	2.22	2.15	2.06	2.17	2.18
Late infant mortality		4.00	3.83	3.77	3.70	3.61	3.68	3.58	3.23	3.42	3.24
Infant mortality		12.60	12.50	11.90	11.61	11.54	11.57	11.46	10.80	11.08	10.92

Source: *Sistema de Informações de Mortalidade* (SIM – Mortality Information System) and *Sistema de Informação sobre Nascidos Vivos* (Sinasc – Live Birth Information System) (Brasil, 2008).

The IMR showed a significant reduction trend between 2008 and 2017, with APC of −1.60% (95%CI −2.06 to −1.13). ENIM showed less intense reduction (APC: −1.18%; 95%CI −1.63 to −0.72), followed by LNIM (APC: −1.76%; 95%CI −2.77 to −0.74) and IMR (APC: −2.12%; 95% CI −2.79 to −1.45), with the greatest reduction ( Chart 2 and Figure ).

**Chart 2:** Distribution of the annual percentage change in infant mortality rates according to age components and according to groups and subgroups of preventable causes of death by interventions of the Brazilian Unified Health System for early neonatal infant mortality, state of São Paulo, from 2008 to 2017.

Child mortality component	Preventable causes of death	Period	APC	Lower 95%CI	Higher 95%CI	P	AAPC	Lower 95%CI	Higher 95%CI
	1.1. Reducible by immunoprevention actions	2008–2017							
	1.2.1. Reducible by adequate attention to the woman during pregnancy	2008–2017	−1.03	−1.92	−0.13	0.03			
	1.2.2. Reducible by adequate attention to the woman during delivery a	2008–2015	−2.31	−4.94	0.41	0.08	−0.09	−3.84	3.82
	1.2.2. Reducible by adequate attention to the woman during delivery a	2015–2017	8.09	−11.94	32.67	0.37			
	1.2.3. Reducible by adequate attention to the fetus and the newborn	2008–2017	−2.6	−4.07	−1.11	0.00			
	1.3. Reducible by actions of diagnosis and adequate treatment	2008–2017	−10.95	−22.25	1.99	0.08			
	1.4. Reducible by adequate health promotion actions	2008–2017	−2.74	−8.71	3.63	0.34			
	2. Ill-defined causes b	2008–2011	−16.44	−30.00	−0.25	0.05	−2.72	−7.86	2.72
	2. Ill-defined causes b	2011–2017	4.97	−1.13	11.45	0.09			
	3. Other causes (not clearly preventable)	2008–2017	−0.24	−1.33	0.85	0.62			
Early neonatal infant mortality	Total	2008–2017	−1.18	−1.63	−0.72	0			
Late neonatal infant mortality	Total	2008–2017	−1.76	−2.77	−0.74	0			
Late infant mortality	Total	2008–2017	−2.12	−2.79	−1.45	0			
Infant mortality	Total	2008–2017	−1.60	−2.06	−1.13	0			

a Joinpoint in 2015.

b Joinpoint in 2011.

Source: *Sistema de Informações de Mortalidade* (SIM – Mortality Information System) and *Sistema de Informação sobre Nascidos Vivos* (Sinasc – Live Birth Information System) (Brasil, 2008).

APC: annual percentage change; AAPC: average annual percentage change.

**Figure:**
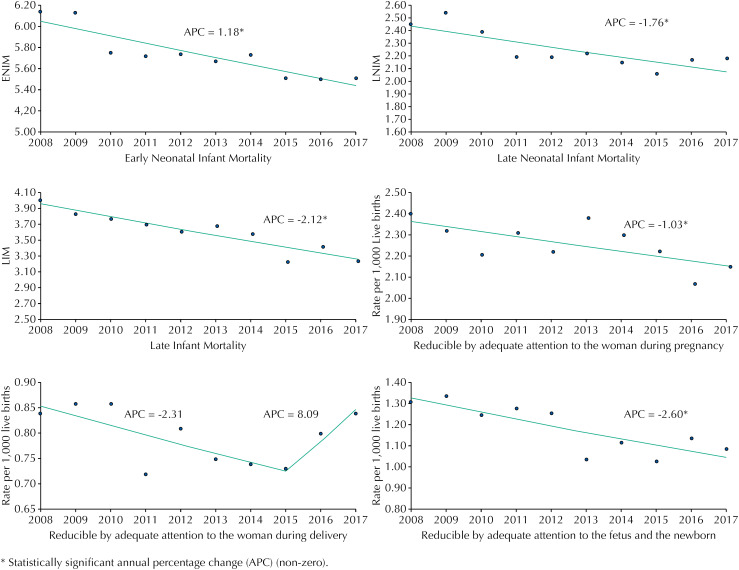
Trend curves of infant mortality rates by age component and early neonatal infant mortality rates by subgroup of preventable causes, State of São Paulo, 2008 to 2017.

Chart 2 also presents the trends in the rates of these deaths with less than seven days of life and their APC, according to the groups and subgroups that constitute the Brazilian list of preventable causes of death by SUS interventions. The subgroup of causes reducible by immunoprevention actions presented only three deaths in the period, thus, it was not possible to establish a trend line.

The three subgroups of causes related to adequate attention to women during pregnancy, adequate attention to women during delivery and adequate attention to the fetus and newborn were responsible for most deaths in children up to the sixth day of life, representing 72% to 75% of the total of these deaths in the period studied.

In the first subgroup, the APC showed a significant decrease, being −1.03%, with 95%CI from −1.92 to −0.13 ( Figure ). In the subgroup of causes reducible by adequate attention to women during delivery, the curve presented an inflection point (joinpoint) in 2015. Thus, a first segment was identified, with a decrease between 2008 and 2015 (APC: −2.31%; 95%CI −4.94 to 0.41), and a second segment, with an increase between 2015 and 2017 (APC: 8.09%; 95%CI −11.94 to 32.67). These trends did not show statistically significant results in any period. The APC for the total period (2008 to 2017) was 0.09%, with 95%CI of −3.84 to 3.82 ( Figure ). The subgroup of causes reducible by adequate attention to the fetus and the newborn presented the largest annual reduction (APC: −2.60; 95%CI −4.07 to −1.11).

The subgroups of causes related to diagnosis and adequate treatment and health promotion actions presented little expression in the set of early neonatal deaths, with APC without statistical significance. The group of undefined causes, also little expression in the set of early neonatal deaths, showed a reduction trend between 2008 and 2011 (APC: −16.44%; 95%CI −30.00 to −0.25) and a non-significant increase between 2011 and 2017 (APC: 4.97; 95%CI −1.13 to 11.45). The APC of the total period was −2.72 (95%CI −7.86 to 2.72). The group of other causes (not clearly preventable) participated with 22% to 24% of the total early neonatal deaths, with no significant trend of variation in rates ( Chart 2 ).

Chart 3 shows the distribution of APC according to the RDH and the municipality of São Paulo for the three subgroups of preventable causes of death with greater magnitudes. In the subgroup of causes reducible by adequate attention to women during pregnancy, death rates show important differences between the various RDH. The APCs presented distinct behavior among the RDH. The RDHs of Taubaté and São José do Rio Preto presented a trend of reduction in the rates of this subgroup of causes: the first with a high initial rate and above the value for the state of São Paulo (3.57 deaths per 1,000 live births) and a significant reduction in 2017, with APC of −5.56 (95%CI −9.60 to 1.33), and the last with an initial rate of 2.01 deaths per 1,000 live births, lower than verified in the state of São Paulo, and APC of −4.82 (95%CI −8.64 to 0.84). The municipality of São Paulo showed an increase in the rate until 2015 (APC: 2.82; 95%CI 0.61 to 5.07) and a decrease between 2015 and 2017, without significant APC. The AAPC for the total period did not show a significant trend.

**Chart 3:** Distribution of the annual percentage change in early neonatal infant mortality rates by Regional Department of Health (RDH), in the selected subgroups of preventable causes of death, state of São Paulo, for the years 2008 and 2017.

Subgroups of preventable causes of death	RDH	ENIM rate		Trend						
		2008	2017	Period	APC	Lower 95%CI	Higher 95%CI	AAPC	Lower 95%CI	Higher 95%CI
1.2.1. Reducible by adequate attention to the woman during pregnancy	Araçatuba	2.55	2.48	2008–2017	−0.76	−4.55	3.18			
	Araraquara	4.10	2.13	2008–2017	−4.58	−9.36	0.46			
	Baixada Santista	2.86	2.90	2008–2017	−1.90	−4.26	0.52			
	Barretos	2.46	3.90	2008–2017	5.84	−0.96	13.1			
	Bauru	2.58	2.19	2008–2017	−3.31	−7.63	1.22			
	Campinas	2.00	1.69	2008–2017	−3.16	−6.99	0.83			
	Franca	2.68	2.27	2008–2017	−3.57	−8.20	1.28			
	Grande São Paulo (excludes MSP)	2.41	2.08	2008–2017	−1.17	−2.78	0.48			
	Marília	2.75	2.75	2008–2017	−1.25	−7.09	4.97			
	Piracicaba	1.81	2.42	2008–2017	0.37	−5.34	6.43			
	Presidente Prudente	2.01	3.51	2008–2017	2.18	−6.09	11.18			
	Registro	2.84	3.02	2008–2017	3.62	−3.49	11.24			
	Ribeirão Preto	2.32	2.17	2008–2012	10.77	−6.19	30.8	−1.40	−8.54	6.30
	Ribeirão Preto			2012–2017	−10.17	−20.13	1.03			
	São João da Boa Vista	2.76	2.84	2008–2017	−3.28	−8.63	2.37			
	São José do Rio Preto	2.01	1.41	2008–2017	−4.82	−8.64	−0.84			
	São Paulo (municipality)	2.09	1.91	2008–2015	2.82	0.61	5.07	−0.44	−3.42	2.64
	São Paulo (municipality)			2015–2017	−11.04	−24.38	4.67			
	Sorocaba	2.67	2.52	2008–2017	2.24	−1.03	5.62			
	Taubaté	3.57	2.41	2008–2017	−5.56	−9.60	−1.33			
1.2.2. Reducible by adequate attention to the woman during delivery	Araçatuba	1.66	1.19	2008–2017	−1.47	−15.56	14.97			
	Araraquara	0.60	0.49	2008–2015	10.1	2.53	18.23	−3.10	−12.29	7.06
	Araraquara	2015–2017	−38.02	−63.63	5.61					
	Baixada Santista	1.51	0.66	2008–2017	−6.64	−10.53	−2.57			
	Barretos	0.38	0.74	2008–2017	2.64	−9.50	16.40			
	Bauru	1.20	1.03	2008–2017	−0.06	−5.77	5.99			
	Campinas	0.62	0.85	2008–2017	−2.05	−7.52	3.75			
	Franca	0.64	0.54	2008–2017	−2.69	−11.2	6.64			
	Grande São Paulo (excludes MSP)	0.96	0.77	2008–2017	−1.48	−4.06	1.17			
	Marília	1.56	0.87	2008–2017	−4.35	−11.94	3.89			
	Piracicaba	0.80	0.47	2008–2017	−6.10	−13.35	1.76			
	Presidente Prudente	1.23	1.60	2008–2017	6.65	−3.19	17.48			
	Registro	0.95	2.26	2008–2017	0.92	−12.56	16.48			
	Ribeirão Preto	0.85	0.72	2008–2017	0.48	−6.23	7.68			
	São João da Boa Vista	0.92	0.91	2008–2017	−3.82	−11.66	4.71			
	São José do Rio Preto	0.40	0.38	2008–2017	−1.33	−6.55	4.19			
	São Paulo (municipality)	0.60	0.95	2008–2015	−1.55	−4.17	1.14	4.90	1.01	8.93
	São Paulo (municipality)	2015–2017	30.98	7.04	60.28					
	Sorocaba	0.93	0.85	2008–2017	−3.66	−9.24	2.26			
	Taubaté	0.86	0.83	2008–2017	−2.86	−8.64	3.28			
1.2.3. Reducible by adequate attention to the fetus and the newborn	Araçatuba	1.55	1.40	2008–2017	−1.63	−13.27	11.57			
	Araraquara	1.03	0.49	2008–2017	0.66	−7.62	9.68			
	Baixada Santista	1.23	0.99	2008–2017	0.23	−5.70	6.53			
	Barretos	1.33	1.30	2008–2017	3.45	−4.95	12.60			
	Bauru	1.15	1.54	2008–2017	2.39	−0.79	5.67			
	Campinas	1.12	1.09	2008–2017	−0.34	−1.87	1.22			
	Franca	0.54	0.87	2008–2017	0.46	−8.67	10.50			
	Grande São Paulo (excludes MSP)	1.32	1.05	2008–2017	−3.14	−5.69	−0.51			
	Marília	1.78	1.52	2008–2017	0.30	−7.04	8.21			
	Piracicaba	0.85	0.95	2008–2017	−0.98	−6.05	4.37			
	Presidente Prudente	0.67	1.17	2008–2017	−5.18	−14.74	5.44			
	Registro	0.95	0.75	2008–2014	18.64	−4.87	47.96	0.10	−18.08	22.32
	Registro	2014–2017	−28.73	−62.92	36.98					
	Ribeirão Preto	0.73	0.78	2008–2017	−1.43	−11.65	9.97			
	São João da Boa Vista	1.33	1.01	2008–2017	−1.25	−6.73	4.56			
	São José do Rio Preto	1.38	0.81	2008–2017	2.37	−6.50	12.09			
	São Paulo (municipality)	1.46	1.07	2008–2017	−9.76	−15.46	−3.66	−4.12	−12.50	5.06
	São Paulo (municipality)	2008–2017	18.5	−27.32	93.22					
	Sorocaba	1.93	1.32	2008–2017	−4.63	−7.67	−1.50			
	Taubaté	1.23	1.28	2008–2017	0.48	−3.29	4.04			

Source: *Sistema de Informações de Mortalidade* (SIM – Mortality Information System) and *Sistema de Informação sobre Nascidos Vivos* (Sinasc – Live Birth Information System) (Brazil, 2008).

APC: annual percentage change; AAPC: average annual percentage change; MSP: municipality of São Paulo.

In the subgroup of reducible causes due to adequate attention to women during delivery, the RDH of Baixada Santista stands out for its reduction trend, with APC of −6.64 (95%CI −10.53 to −2.57). From 2008 to 2015, the municipality of São Paulo showed a non-significant reduction trend (APC −1.55; 95%CI −4.17 to 1.14), followed by a strong increase in the period from 2015 to 2017 (APC: 30.98; 95%CI 7.04 to 60.28), resulting in an AAPC of 4.90 (95%CI 1.01 to 8.93) between 2008 and 2017. The Araraquara region showed an increase in this rate between 2008 and 2015 (APC: 10.10; 95%CI 2.53 to 18.23) and a non-significant decrease in the following years (APC: −38.02; 95%CI −63.63 to 5.61). The AAPC for the entire period did not show significant results.

In the subgroup of reducible causes due to adequate attention to the fetus and the newborn, the municipality of São Paulo showed a significant reduction between 2008 and 2015 (APC: −9.76; 95%CI −15.46 to −3.66) and a non-significant increase between 2015 and 2017 (APC: 18.50; 95%CI −27.52 to 93.22). The Sorocaba region also showed a significant reduction in the rate (APC: −4.63; 95%CI −7.67 to −1.50). The Greater São Paulo region also showed a significant reduction in rates over the period, with APC of −3.14 (95%CI −5.69 to −0.51).

## DISCUSSION

In the state of São Paulo, the IMR and rates in its three age components showed a consistent downward trend throughout the study period. The IMR presented the largest annual percentage reduction, and ENIM, the smallest reduction. The percentage of deaths before the 7º day represented approximately 50% of the total.

ENIM according to the causes of preventability showed a reduction trend in the subgroups related to attention to women during pregnancy and attention to the fetus and the newborn; however, there was no reduction trend in deaths related to attention to women during delivery. There was no reduction trend among other causes (not clearly preventable), whose reduction depends less on SUS interventions.

When analyzing the ENIM rate according to the RDH, heterogeneity was found in the rates observed in 2008 and 2017, as well as in the APCs. The municipality of São Paulo stands out, showing an inflection point in 2015 in the three trend curves of the subgroups analyzed; in the attention to women during delivery, there was a high and significant trend of increasing rates from 2015, resulting in an increase trend for the entire period. Equivalent behavior was observed in this municipality in relation to the reducible causes by attention to the fetus and the newborn, although without statistical significance.

In a study considering the period from 2006 to 2010, Almeida et al. ^13^ found an early neonatal mortality rate of 6.2 per thousand live births in the state of São Paulo. This points to a significant reduction in early neonatal mortality in the state over the past 10 years. In another study in the state of São Paulo, from 1996 to 2012 ^14^ , the proportion of early neonatal deaths of about 50% of infant deaths was found, showing that this relationship has remained approximately constant for more than fifteen years. In Brazil, there is also this same proportion of early neonatal deaths ^12^ .

Lawn et al. ^15^ , in the world panorama of infant mortality in the year 2000, pointed out that ¾ of neonatal deaths occurred in the first week of life. In the previous 20 years, there was a significant reduction in IMR and a less intense reduction, of only 25%, in neonatal mortality, with little significant progress in ENIM. Moreover, for countries with a high Development Index, the neonatal mortality rate per thousand live births was four deaths; in the region of the Americas, it was three times more ^15^ .

In the analysis of preventability, 75% of early neonatal deaths were attributed to preventable causes related to the attention to women during pregnancy, delivery, and attention to the fetus and newborn. This first of the three subgroups of preventable causes was responsible for most of the deaths and showed a clear trend of reduction. This result may be due to the increase in access to prenatal care, since there was a progressive increase in the coverage of family health teams in the state between 2008 and 2017: from 26.4% to 39.4% ^16^ .

The second subgroup of deaths, reducible by adequate attention to women during delivery, did not show a reduction in rates during the period. In attention during delivery, the establishment of a maternal and child health care network stood out since 2011 ( *Rede Cegonha* ) ^17^ ; however, in implementation evaluations, it was difficult to implement and qualify care processes in different regions of the country ^18^^–^^20^ .

The subgroup of preventable causes reducible by adequate attention to the fetus and newborn presented a significant reduction trend. For this subgroup, a further analysis would be important, considering the potential impact of the *Rede Cegonha*^17^ on the expansion of care offerings aimed at the newborn.

The group of other causes (not clearly preventable) represented 22% to 24% of the total early neonatal deaths, with no significant trend of variation in rates. In a nationwide study conducted by Malta et al. ^21^ between 1977 and 2006, considering all infant deaths, there was also no significant reduction in gross rates, but a significant reduction after adjustment for ill-defined causes.

A study analyzing infant mortality in Brazil from 2000 to 2010 ^22^ points, in ENIM, to the need to strengthen attention to pregnant women, delivery and newborns. Among deaths occurring in the first week of life, low birth weight and prematurity appear as the most prominent factors. Although an important proportion of premature births is not yet preventable, the relationship with smoking, urinary tract infection or asymptomatic bacteriuria, which are amenable to intervention in prenatal care, as factors for reducing prematurity, has already been established. Another point to be considered as a factor for prematurity (and eventual death) is “iatrogenic prematurity due to improper termination of pregnancy” ^23^ in a scenario, in which cesarean delivery rates have remained high ^4^ .

In the comparison between the regions of the state, the heterogeneity in the behavior of rates and their temporal trends is evident. In a study on preventable causes of neonatal death in the state of São Paulo, Nascimento et al. ^23^ observed heterogeneity in the distribution of neonatal deaths in the different regions of the state of São Paulo; considering deaths from preventable causes, they pointed to some regions that should receive more attention.

In deaths reducible by adequate attention to women during pregnancy, despite the trend of reduction in mortality in 11 of the 18 regions studied, these trends presented statistical significance only in two regions. Although there are no systematized data on the quality of prenatal care offered in the different regions of the state of São Paulo, the population coverage data of the Family Health Strategy and primary care as a whole, obtained from the *e-Gestor Atenção Básica*^16^ , show important differences in the coverage of the strategy, which is higher in the western and southern regions of the state, as well as in the municipalities with a smaller population, decreasing in the larger municipalities.

It is worth remembering that the Family Health Strategy teams are composed nuclearly of family health physician, nurse, nursing assistant or technician and community health agents. On the other hand, the other teams, called “traditional,” usually have physicians in the specialties of Pediatrics, Gynecology/Obstetrics and Medical Clinic, as well as nurse and nursing assistant or technician. The different compositions and professional profiles existing between these two types of teams can produce distinct results in the attention to pregnant women.

However, the behavior of early neonatal mortality rates preventable by adequate attention to women during pregnancy is not related to the supply (population coverage) of primary care in the family health modality. An ecological study on the effectiveness of the strategy in child health indicators in the state of São Paulo ^24^ found no relationship between family health coverage and neonatal infant mortality rates; a coverage over 50% showed a protective effect only for the post-neonatal component of infant mortality. Regarding early neonatal deaths preventable by adequate attention to women during delivery, attention is drawn to the unfavorable evolution in the city of São Paulo, which accounts for about 28% of live births in the state, although most regions have achieved significant reduction in their rates.

A final point to be considered is that concerning the classification of preventability used. Dias et al. ^25^ show results of analysis of the preventability of infant deaths using comparatively different classification methods, drawing attention to the need for periodic updating of these classifications in the face of permanent technological changes in attention.

The grouping of causes of death allowed to visualize the influence of the actions of the health services on ENIM. When considering only deaths before the seventh day of life, the participation of determinants related to access and quality of health actions should be predominant, although other factors may also contribute to these deaths. If the analysis of trends points to a probable improvement in access and / or quality of attention to pregnant women and newborns, the analysis of grouped causes of death does not allow to visualize possible intervention points in health services to support these trends, as well as to cope with preventable deaths due to adequate attention to women during delivery, which did not show a significant reduction in the period.
