# Supplementary material for: Trend of preventable deaths up to the 6th day of life in the state of São Paulo – 2008 to 2017
Source: Rev Saude Publica. 2020 Nov 27;54:132. doi: 10.11606/s1518-8787.2020054002309 (PMC7703530; doi:10.11606/s1518-8787.2020054002309)
Supplement: Translation

# Translation: Tendência das mortes evitáveis até o 6º dia de vida no estado de São Paulo – 2008 a 2017

**Authors:** Arnaldo Sala, Carla Gianna Luppi

**Affiliations:** I São PauloSP Brasil Secretaria de Estado da Saúde. Coordenadoria de Regiões de Saúde/Atenção Básica. São Paulo, SP, Brasil; II São PauloSP Brasil Universidade Federal de São Paulo. Escola Paulista de Medicina. Departamento de Medicina Preventiva. São Paulo, SP, Brasil

**Keywords:** Mortalidade Neonatal Precoce, Tocologia, Atenção Integrada às Doenças Prevalentes na Infância, Serviços de Saúde Materno-Infantil, Estudos Ecológicos

## Abstract

**OBJETIVO:**

Analisar a tendência da mortalidade infantil neonatal precoce no estado de São Paulo segundo evitabilidade e região de residência.

**MÉTODO:**

Estudo ecológico com dados secundários de 2008 a 2017, obtidos a partir do Sistema de Informação sobre Nascidos Vivos e do Sistema de Informação sobre Mortalidade. As causas de óbito foram classificadas segundo grupos de evitabilidade, sendo estimadas as variações percentuais anuais das taxas de óbito de cada grupo de evitabilidade, por meio do *software Joinpoint* .

**RESULTADOS:**

O componente neonatal precoce apresentou tendência de redução com variação percentual anual de −1,18 (IC95% −1,63 – −0,72), menos acentuado que os demais componentes etários da mortalidade infantil. Na análise segundo evitabilidade, as causas reduzíveis por atenção à mulher na gestação e as reduzíveis por cuidado ao feto e ao recém-nascido apresentaram variação percentual anual, respectivamente de −1,03 (IC95%: −1,92 – −0,13) e −2,6 (IC95%: −4,07 – −1,11). Nas causas reduzíveis por atenção à mulher no parto, não se observou tendência de redução. Ocorreram discrepâncias regionais na variação das taxas de mortalidade infantil neonatal precoce segundo evitabilidade.

**CONCLUSÕES:**

A mortalidade até o 6º dia de vida apresentou maior dificuldade de redução na comparação com os outros componentes etários. A ausência de tendência de redução nos óbitos evitáveis pelo cuidado à mulher no parto aponta para possível fragilidade na atenção ao parto.

## INTRODUÇÃO

A redução das taxas de mortalidade infantil (TMI) tem sido objeto de preocupação de gestores e formuladores de políticas em saúde. O Brasil cumpriu a meta estabelecida nos objetivos de desenvolvimento do milênio da Organização das Nações Unidas em relação à TMI ^1^ . Em 2015 foi estabelecida a Agenda 2030 para o Desenvolvimento Sustentável ^2^ , na qual se reafirma a preocupação com a redução das mortes de crianças menores de cinco anos, com destaque para a mortalidade neonatal ^3^^,^^4^ . Nesse contexto o acesso aos cuidados de saúde com qualidade torna-se primordial, apontando para a redução das mortes materna, infantil e neonatal, mitigando as mortes evitáveis.

No Brasil, a TMI tem apresentado decréscimo nas últimas décadas, com 29,0 óbitos por 1.000 nascidos vivos em 2000, reduzindo para 17,2 óbitos por 1.000 nascidos vivos em 2010 e alcançando 12,8 óbitos por 1.000 nascidos vivos em 2017 ^5^ . A TMI no estado de São Paulo também tem apresentado uma consistente redução ao longo das últimas décadas, com 16,07 óbitos por 1.000 nascidos vivos no ano de 2001, reduzindo para 11,86 óbitos por mil nascidos vivos em 2010 e alcançando 10,90 óbitos por 1.000 nascidos vivos em 2017 ^6^ .

A mortalidade neonatal e também a neonatal precoce (óbitos com menos de sete dias de vida) apresentaram redução importante na década passada no estado de São Paulo. No entanto, essa redução foi menos acentuada no período entre 2011 e 2017: de 7,90 para 7,66 óbitos neonatais por 1.000 nascidos vivos em 2017 ^6^ .

Os óbitos infantis até o 27º dia de vida corresponderam a cerca de 68% a 70% do total dos óbitos infantis neste mesmo período. Considerando somente aqueles ocorridos até o 6º dia de vida, tem-se cerca de 49% a 51% do total de óbitos infantis ^6^ .

Em estudo de revisão da mortalidade infantil no Brasil, Santos et al. ^7^ apontam para a mudança ocorrida no período compreendido entre 1980 e 1983 e no período mais recente de 2005 a 2008, em que se destacam os fatores relacionados ao recém-nascido, maternos, assistenciais e socioeconômicos. Embora parcela relevante dos fatores associados à mortalidade neonatal esteja associada a aspectos biológicos e sociodemográficos do recém-nascido ^8^^,^^9^ , é importante considerar a questão da evitabilidade desses óbitos, tendo como referência o acesso e a qualidade dos recursos assistenciais disponíveis para o acompanhamento durante a gestação, assim como os recursos disponíveis para a atenção ao parto e ao recém-nascido.

Malta e Duarte ^10^ consideram a classificação de evitabilidade, ou de causa de morte evitável, quando o óbito é passível de prevenção por ação dos serviços de saúde. No Brasil, foi publicada em 2008 uma lista de causas de mortes evitáveis por intervenções do Sistema Único de Saúde (SUS), posteriormente atualizada ^11^ .

Como ainda persistem desafios para a redução da mortalidade infantil e, particularmente, a neonatal precoce, este estudo pretende analisar as tendências das taxas de mortalidade infantil neonatal precoce (MINP) segundo a classificação de causas de mortes evitáveis por intervenções do SUS para o estado de São Paulo e verificar o comportamento destas taxas em cada uma das regiões administrativas que correspondem aos departamentos regionais de saúde (DRS) da Secretaria de Estado da Saúde de São Paulo.

## MÉTODOS

Trata-se de estudo ecológico de série temporal das taxas de mortalidade infantil que tem como universo o total dos óbitos infantis ocorridos no estado de São Paulo, no período compreendido entre os anos de 2008 e 2017.

A fonte de dados dos óbitos infantis foi a base de dados do Sistema de Informação de Mortalidade (SIM) disponível para download no site do Datasusᵃ. Os dados dos nascidos vivos eram provenientes do Sistema de Informações de Nascidos Vivos (Sinasc), consolidados no site do Datasus, em Informações de Saúde (Tabnet), subseção Estatísticas Vitais ^12^ . O ano de 2017 era o mais recente disponível no site do Datasus; deste modo, para um estudo de série temporal, foram considerados os últimos 10 anos disponíveis.

As variáveis incluídas na análise foram: idade do óbito, causa básica do óbito e o DRS de residência do indivíduo. A idade do óbito foi considerada para identificação dos três componentes etários da mortalidade infantil: MINP de 0 a 6 dias, mortalidade infantil neonatal tardia (MINT) de 7 a 27 dias e mortalidade infantil tardia (MIT) de 28 a 364 dias.

A causa básica do óbito codificada pela Classificação Internacional de Doenças (CID) foi agrupada segundo a Lista Brasileira de Causas de Morte Evitáveis por Intervenções do SUS para a faixa etária de 0 a 4 anos ^11^ . Essa lista é composta por três grupos de causas: grupo 1 – causas evitáveis (dividido em 6 subgrupos); grupo 2 – causas de morte maldefinidas; grupo 3 – demais causas (não claramente evitáveis).

Os municípios de residência foram agrupados segundo as áreas de abrangência dos DRS que compõem a estrutura administrativa da Secretaria de Estado da Saúde de São Paulo. O município de São Paulo foi destacado do DRS da Grande São Paulo em virtude de sua magnitude populacional, o que resulta em um total de 18 regiões.

Foram calculadas as taxas de óbito por 1.000 nascidos vivos para cada ano compreendido neste estudo, bem como as taxas para cada um dos três componentes etários. Para a análise específica dos óbitos neonatais precoces, foram calculadas as taxas de óbito específicas para cada grupo e subgrupo de causas de morte evitáveis.

Os dados foram inicialmente tabulados por meio do software IBM SPSS Statistics 20. Os cálculos das taxas de mortalidade foram realizados utilizando-se o software Microsoft Excel, versão 2010.

Foram analisadas as tendências das taxas de mortalidade infantil em cada componente etário. Para a MINP foram analisadas as tendências das taxas específicas para cada grupo e subgrupo de causas de morte evitáveis. Na análise da MINP nas áreas dos DRS, somente foram analisadas as tendências para os três subgrupos de evitabilidade com maior relevância para as mortes ocorridas até o 6º dia de vida: reduzíveis por adequada atenção à mulher na gestação, reduzíveis por adequada atenção à mulher no parto e reduzíveis por adequada atenção ao feto e recém-nascido.

A análise das tendências foi realizada calculando-se as estimativas de variação percentual anual ( *annual percent change* [APC]) das taxas de mortalidade no período de 2008 a 2017, utilizando-se o software *Joinpoint* Regression Program 4.7.0.0, adotando-se modelo log-linear com o ano do óbito como variável independente e as diversas taxas como variáveis dependentes. O software identifica os eventuais pontos de inflexão ( *joinpoint* ) na curva de tendência, quando ocorre mudança significativa na inflexão da curva. Na ocorrência de *joinpoint,* é calculada também a variação percentual anual média para o período ( *average annual percent change* [AAPC]). Foram calculados os intervalos de confiança (IC) para 95%.

A base de dados utilizada é disponível para acesso público em http://datasus.saude.gov.br/informacoes-de-saude/servicos2/transferencia-de-arquivos e não possui nenhum campo com dados identificando as pessoas individualmente. Por ser banco de dados de acesso público sem identificação individual, nos termos da Lei nº 12.527, de 18 de novembro de 2011, não foi necessária a avaliação por comitê de ética em pesquisa, em acordo com a Resolução nº 510/2016 do Conselho Nacional de Saúde.

## RESULTADOS

Nos anos de 2008 e 2017, foram registrados 70.858 óbitos infantis, sendo 35.071 ocorridos até o 6º dia de vida, 13.767 ocorridos entre o 7º e o 27º dia de vida e 22.020 ocorridos com 28 ou mais dias de vida. A TMI variou de 12,60 em 2008 para 10,92 óbitos por 1.000 nascidos vivos em 2017. A MINP participou com 48,8% dos óbitos em 2008 para 50,4% em 2017. As taxas de MINP variaram de 6,14 em 2008 para 5,51 óbitos por 1.000 nascidos vivos em 2017 ( Quadro 1 ).

**Quadro 1:** Taxas de mortalidade infantil segundo componentes etários e segundo os grupos e subgrupos das causas de morte evitáveis por intervenções do Sistema Único de Saúde para a mortalidade infantil neonatal precoce, estado de São Paulo, anos de 2008 a 2017.

Componente/causas de morte evitáveis		Ano do óbito									
		2008	2009	2010	2011	2012	2013	2014	2015	2016	2017
Mortalidade infantil neonatal precoce		6,14	6,13	5,75	5,72	5,74	5,67	5,73	5,51	5,50	5,51
	1.1. Reduzíveis por ações de imunoprevenção	0,00	0,00	–	–	–	–	–	–	–	–
	1.2.1. Reduzíveis por adequada atenção à mulher na gestação	2,40	2,32	2,21	2,31	2,22	2,38	2,30	2,22	2,07	2,15
	1.2.2. Reduzíveis por adequada atenção à mulher no parto	0,84	0,86	0,86	0,72	0,81	0,75	0,74	0,73	0,80	0,84
	1.2.3. Reduzíveis por adequada atenção ao feto e ao recém-nascido	1,31	1,34	1,25	1,28	1,26	1,04	1,12	1,03	1,14	1,09
	1.3. Reduzíveis por ações de diagnóstico e tratamento adequado	0,02	0,02	0,01	0,01	0,01	0,00	0,01	0,02	0,00	0,01
	1.4. Reduzíveis por ações de promoção à saúde vinculadas à ações de atenção à saúde	0,03	0,03	0,02	0,03	0,03	0,04	0,02	0,02	0,03	0,02
	2. Causas maldefinidas	0,20	0,17	0,17	0,10	0,12	0,16	0,15	0,15	0,14	0,16
	3. Demais causas (não claramente evitáveis)	1,35	1,38	1,23	1,28	1,30	1,30	1,39	1,34	1,32	1,24
Mortalidade infantil neonatal tardia		2,45	2,54	2,39	2,19	2,19	2,22	2,15	2,06	2,17	2,18
Mortalidade infantil tardia		4,00	3,83	3,77	3,70	3,61	3,68	3,58	3,23	3,42	3,24
Mortalidade infantil		12,60	12,50	11,90	11,61	11,54	11,57	11,46	10,80	11,08	10,92

Fonte: Sistema de Informações de Mortalidade (SIM) e Sistema de Informações de Nascidos Vivos (Sinasc) (Brasil, 2008).

A TMI apresentou tendência de redução significativa no período entre 2008 e 2017, com APC de −1,60% (IC95% −2,06 – −1,13). A MINP apresentou redução menos intensa (APC: −1,18%; IC95% −1,63 – −0,72), seguida pela MINT (APC: −1,76%; IC95% −2,77 – −0,74) e a MIT (APC: −2,12%; IC95% −2,79 – −1,45), com a maior redução ( Quadro 2 e Figura ).

**Quadro 2:** Distribuição da variação percentual anual das taxas de mortalidade infantil segundo componentes etários e segundo grupos e subgrupos das causas de morte evitáveis por intervenções do Sistema Único de Saúde para a mortalidade infantil neonatal precoce, estado de São Paulo, anos de 2008 a 2017.

Componente da mortalidade infantil	Causas de morte evitável	Período	APC	IC95% inferior	IC95% superior	P	AAPC	IC95% inferior	IC95% superior
	1.1. Reduzíveis por ações de imunoprevenção	2008–2017							
	1.2.1. Reduzíveis por adequada atenção à mulher na gestação	2008–2017	−1,03	−1,92	−0,13	0,03			
	1.2.2. Reduzíveis por adequada atenção à mulher no parto a	2008–2015	−2,31	−4,94	0,41	0,08	−0,09	−3,84	3,82
	1.2.2. Reduzíveis por adequada atenção à mulher no parto a	2015–2017	8,09	−11,94	32,67	0,37			
	1.2.3. Reduzíveis por adequada atenção ao feto e ao recém-nascido	2008–2017	−2,6	−4,07	−1,11	0,00			
	1.3. Reduzíveis por ações diagnóstico e tratamento adequado	2008–2017	−10.95	−22.25	1.99	0,08			
	1.4. Reduzíveis por ações adequadas de promoção à saúde	2008–2017	−2,74	−8,71	3,63	0,34			
	2. Causas maldefinidas b	2008–2011	−16,44	−30,00	−0,25	0,05	−2,72	−7,86	2,72
	2. Causas maldefinidas b	2011–2017	4,97	−1,13	11,45	0,09			
	3. Demais causas (não claramente evitáveis)	2008–2017	−0,24	−1,33	0,85	0,62			
Mortalidade infantil neonatal precoce		Total	2008–2017	−1,18	−1,63	−0,72	0		
Mortalidade infantil neonatal tardia		Total	2008–2017	−1,76	−2,77	−0,74	0		
Mortalidade infantil tardia		Total	2008–2017	−2,12	−2,79	−1,45	0		
Mortalidade infantil		Total	2008–2017	−1,60	−2,06	−1,13	0		

a *Joinpoint* em 2015.

b *Joinpoint* em 2011.

Fonte: Sistema de Informações de Mortalidade (SIM) e Sistema de Informações de Nascidos Vivos (SINASC) (Brasil, 2008).

APC: variação percentual anual; AAPC: variação percentual anual média

**Figura:** Curvas de tendência das taxas de mortalidade infantil por componente etário e das taxas de mortalidade infantil neonatal precoce por subgrupo de causas evitáveis, estado de São Paulo, 2008 a 2017.

O Quadro 2 apresenta também as tendências das taxas destes óbitos com menos de sete dias de vida e respectivas APC, segundo os grupos e subgrupos que compõem a Lista Brasileira de Causas de Morte Evitáveis por Intervenções do SUS. O subgrupo de causas reduzíveis por ações de imunoprevenção apresentou apenas três óbitos no período, não sendo possível estabelecer linha de tendência.

Os três subgrupos de causas relacionadas à adequada atenção à mulher na gestação, à adequada atenção à mulher no parto e ao adequado cuidado ao feto e ao recém-nascido foram responsáveis pela maior parte dos óbitos em crianças até o sexto dia de vida, participando com 72% a 75% do total desses óbitos no período estudado.

No primeiro subgrupo a APC apresentou decréscimo significativo, ficando em −1,03%, com IC95% de −1,92 a −0,13 ( Figura ). No subgrupo das causas reduzíveis por adequada atenção à mulher no parto, a curva apresentou um ponto de inflexão ( *joinpoint* ) no ano de 2015. Desse modo, ficou identificado um primeiro segmento, com redução no período de 2008 a 2015 (APC: −2,31%; IC95% −4,94 – 0,41), e um segundo segmento, com aumento no período de 2015 a 2017 (APC: 8,09%; IC95% −11,94 – 32,67). Essas tendências não apresentaram resultado estatisticamente significante em nenhum dos períodos. A AAPC do período total (2008 a 2017) foi de 0,09%, com IC95% de −3,84 a 3,82 ( Figura ). O subgrupo das causas reduzíveis por adequado cuidado ao feto e ao recém-nascido apresentou a maior redução anual (APC: −2,60; IC95% −4,07 – −1,11).

Os subgrupos de causas relacionadas a diagnóstico e tratamento adequado e a ações de promoção à saúde apresentaram pouca expressão no conjunto dos óbitos neonatais precoces, com APC sem significado estatístico. O grupo das causas maldefinidas, também pouca expressão no conjunto dos óbitos neonatais precoces, apresentou tendência de redução no período de 2008 a 2011 (APC: −16,44%; IC −30,00 – −0,25) e aumento não significativo no período de 2011 a 2017 (APC: 4,97; IC95% −1,13 – 11,45). A AAPC do período total foi de −2,72 (IC95% −7,86 – 2,72). O grupo das demais causas (não claramente evitáveis) participou com 22% a 24% do total de óbitos neonatais precoces, sem tendência significativa de variação das taxas ( Quadro 2 ).

O Quadro 3 apresenta a distribuição da APC segundo os DRS e o município de São Paulo para os três subgrupos de causas evitáveis de óbito com maiores magnitudes. No subgrupo de causas reduzíveis por adequada atenção à mulher na gestação, as taxas de óbito mostram diferenças importantes entre os diversos DRS. As APC apresentaram comportamento distintos entre os DRS. Apresentaram tendência de redução nas taxas desse subgrupo de causas os DRS de Taubaté e São José do Rio Preto: o primeiro com uma taxa inicial alta e acima do valor para o estado de São Paulo (3,57 óbitos por 1.000 nascidos vivos) e redução importante em 2017, com APC de −5,56 (IC95% −9,60 – 1,33), e o último com taxa inicial de 2,01 óbitos por 1.000 nascidos vivos, inferior à verificada no estado de São Paulo, e APC de −4,82 (IC95% −8,64 – 0,84). O município de São Paulo apresentou tendência de aumento na taxa até 2015 (APC: 2,82; IC95% 0,61 – 5,07) e redução no período de 2015 a 2017, sem APC significativa. A AAPC para o período total não apresentou tendência significativa.

**Quadro 3:** Distribuição da variação percentual anual das taxas de mortalidade infantil neonatal precoce por Departamento Regional de Saúde (DRS), nos subgrupos de causas evitáveis de óbito selecionados, estado de São Paulo, para os anos de 2008 e 2017.

Subgrupos das causas de morte evitáveis	DRS	Taxa de MINP		Tendência						
		2008	2017	Período	APC	IC95% inferior	IC95% superior	AAPC	IC95% inferior	IC95% superior
1.2.1. Reduzíveis por adequada atenção à mulher na gestação	Araçatuba	2,55	2,48	2008–2017	−0,76	−4,55	3,18			
	Araraquara	4,10	2,13	2008–2017	−4,58	−9,36	0,46			
	Baixada Santista	2,86	2,90	2008–2017	−1,90	−4,26	0,52			
	Barretos	2,46	3,90	2008–2017	5,84	−0,96	13,1			
	Bauru	2,58	2,19	2008–2017	−3,31	−7,63	1,22			
	Campinas	2,00	1,69	2008–2017	−3,16	−6,99	0,83			
	Franca	2,68	2,27	2008–2017	−3,57	−8,20	1,28			
	Grande São Paulo (exclui MSP)	2,41	2,08	2008–2017	−1,17	−2,78	0,48			
	Marília	2,75	2,75	2008–2017	−1,25	−7,09	4,97			
	Piracicaba	1,81	2,42	2008–2017	0,37	−5,34	6,43			
	Presidente Prudente	2,01	3,51	2008–2017	2,18	−6,09	11,18			
	Registro	2,84	3,02	2008–2017	3,62	−3,49	11,24			
	Ribeirão Preto	2,32	2,17	2008–2012	10,77	−6,19	30,8	−1,40	−8,54	6,30
	Ribeirão Preto	2012–2017	−10,17	−20,13	1,03					
	São João da Boa Vista	2,76	2,84	2008–2017	−3,28	−8,63	2,37			
	São José do Rio Preto	2,01	1,41	2008–2017	−4,82	−8,64	−0,84			
	São Paulo (município)	2,09	1,91	2008–2015	2,82	0,61	5,07	−0,44	−3,42	2,64
	São Paulo (município)	2015–2017	−11,04	−24,38	4,67					
	Sorocaba	2,67	2,52	2008–2017	2,24	−1,03	5,62			
	Taubaté	3,57	2,41	2008–2017	−5,56	−9,60	−1,33			
1.2.2. Reduzíveis por adequada atenção à mulher no parto	Araçatuba	1,66	1,19	2008–2017	−1,47	−15,56	14,97			
	Araraquara	0,60	0,49	2008–2015	10,1	2,53	18,23	−3,10	−12,29	7,06
	Araraquara	2015–2017	−38,02	−63,63	5,61					
	Baixada Santista	1,51	0,66	2008–2017	−6,64	−10,53	−2,57			
	Barretos	0,38	0,74	2008–2017	2,64	−9,50	16,40			
	Bauru	1,20	1,03	2008–2017	−0,06	−5,77	5,99			
	Campinas	0,62	0,85	2008–2017	−2,05	−7,52	3,75			
	Franca	0,64	0,54	2008–2017	−2,69	−11,2	6,64			
	Grande São Paulo (exclui MSP)	0,96	0,77	2008–2017	−1,48	−4,06	1,17			
	Marília	1,56	0,87	2008–2017	−4,35	−11,94	3,89			
	Piracicaba	0,80	0,47	2008–2017	−6,10	−13,35	1,76			
	Presidente Prudente	1,23	1,60	2008–2017	6,65	−3,19	17,48			
	Registro	0,95	2,26	2008–2017	0,92	−12,56	16,48			
	Ribeirão Preto	0,85	0,72	2008–2017	0,48	−6,23	7,68			
	São João da Boa Vista	0,92	0,91	2008–2017	−3,82	−11,66	4,71			
	São José do Rio Preto	0,40	0,38	2008–2017	−1,33	−6,55	4,19			
	São Paulo (município)	0,60	0,95	2008–2015	−1,55	−4,17	1,14	4,90	1,01	8,93
	São Paulo (município)	2015–2017	30,98	7,04	60,28					
	Sorocaba	0,93	0,85	2008–2017	−3,66	−9,24	2,26			
	Taubaté	0,86	0,83	2008–2017	−2,86	−8,64	3,28			
1.2.3. Reduzíveis por adequada atenção ao feto e ao recém-nascido	Araçatuba	1,55	1,40	2008–2017	−1,63	−13,27	11,57			
	Araraquara	1,03	0,49	2008–2017	0,66	−7,62	9,68			
	Baixada Santista	1,23	0,99	2008–2017	0,23	−5,70	6,53			
	Barretos	1,33	1,30	2008–2017	3,45	−4,95	12,60			
	Bauru	1,15	1,54	2008–2017	2,39	−0,79	5,67			
	Campinas	1,12	1,09	2008–2017	−0,34	−1,87	1,22			
	Franca	0,54	0,87	2008–2017	0,46	−8,67	10,50			
	Grande São Paulo (exclui MSP)	1,32	1,05	2008–2017	−3,14	−5,69	−0,51			
	Marília	1,78	1,52	2008–2017	0,30	−7,04	8,21			
	Piracicaba	0,85	0,95	2008–2017	−0,98	−6,05	4,37			
	Presidente Prudente	0,67	1,17	2008–2017	−5,18	−14,74	5,44			
	Registro	0,95	0,75	2008–2014	18,64	−4,87	47,96	0,10	−18,08	22,32
	Registro	2014–2017	−28,73	−62,92	36,98					
	Ribeirão Preto	0,73	0,78	2008–2017	−1,43	−11,65	9,97			
	São João da Boa Vista	1,33	1,01	2008–2017	−1,25	−6,73	4,56			
	São José do Rio Preto	1,38	0,81	2008–2017	2,37	−6,50	12,09			
	São Paulo (município)	1,46	1,07	2008–2017	−9,76	−15,46	−3,66	−4,12	−12,50	5,06
	São Paulo (município)	2008–2017	18,5	−27,32	93,22					
	Sorocaba	1,93	1,32	2008–2017	−4,63	−7,67	−1,50			
	Taubaté	1,23	1,28	2008–2017	0,48	−3,29	4,04			

Fonte: Sistema de Informações de Mortalidade (SIM) e Sistema de Informações de Nascidos Vivos (Sinasc) (Brasil, 2008).

APC: variação percentual anual; AAPC: variação percentual anual média; MSP: município de São Paulo.

No subgrupo de causas reduzíveis por adequada atenção à mulher no parto destaca-se, pela tendência de redução, o DRS da Baixada Santista, com APC de −6,64 (IC95% −10,53 – −2,57). O município de São Paulo apresentou no período de 2008 a 2015 uma tendência não significativa de redução (APC −1,55; IC95% −4,17 – 1,14), seguida de forte aumento no período de 2015 a 2017 (APC: 30,98; IC95% 7,04 – 60,28), resultando no período de 2008 a 2017 em AAPC de 4,90 (IC95% 1,01 – 8,93). A região de Araraquara apresentou tendência de aumento nessa taxa no período de 2008 a 2015 (APC: 10,10; IC95% 2,53 – 18,23) e redução não significativa nos anos seguintes (APC: −38,02; IC95% −63,63 – 5,61). A AAPC do período inteiro não apresentou resultado significativo.

No subgrupo de causas reduzíveis por adequada atenção ao feto e ao recém-nascido, o município de São Paulo apresentou redução significativa no período de 2008 a 2015 (APC: −9,76; IC95% −15,46 – −3,66) e aumento não significativo no período de 2015 a 2017 (APC: 18,50; IC95% −27,52 – 93,22). A região de Sorocaba também apresentou tendência de redução significativa da taxa (APC: −4,63; IC95% −7,67 – −1,50). A região da Grande São Paulo também apresentou tendência significativa de redução das taxas ao longo do período, com APC de −3,14 (IC95% −5,69 – −0,51).

## DISCUSSÃO

No estado de São Paulo, a TMI e as taxas nos seus três componentes etários apresentaram consistente tendência de redução em todo o período estudado. A MIT apresentou a maior redução percentual anual, e a MINP a menor redução. O percentual de óbitos antes do 7º dia representou aproximadamente 50% do total.

Na MINP segundo as causas de evitabilidade, observou-se tendência de redução nos subgrupos relacionados à atenção a mulher na gestação e atenção ao feto e ao recém-nascido; entretanto, não houve tendência de redução dos óbitos relacionadas à atenção a mulher no parto. Não foi observada tendência de redução entre as demais causas (não claramente evitáveis), cuja redução depende menos das intervenções do SUS.

Ao se analisar a taxa de MINP segundo os DRS, encontrou-se heterogeneidade nas taxas observadas em 2008 e 2017, assim como nas APC. Destaca-se o município de São Paulo, que apresentou ponto de inflexão em 2015 nas três curvas de tendência dos subgrupos analisados; na atenção à mulher no parto ocorreu uma elevada e significativa tendência de aumento das taxas a partir de 2015, resultando em tendência de aumento para o período inteiro. Comportamento equivalente foi observado nesse município em relação às causas reduzíveis pela atenção ao feto e ao recém-nascido, embora sem significância estatística.

Em estudo considerando o período de 2006 a 2010, Almeida et al. ^13^ encontraram taxa de mortalidade neonatal precoce de 6,2 por mil nascidos vivos no estado de São Paulo. Isso aponta para uma redução importante da mortalidade neonatal precoce no estado nos últimos 10 anos. Em outro estudo no estado de São Paulo, no período de 1996 a 2012 ^14^ , foi encontrada proporção de óbitos neonatais precoces de cerca de 50% dos óbitos infantis, mostrando que essa relação vem se mantendo aproximadamente constante há mais de uma década e meia. No Brasil, também se verifica essa mesma proporção de óbitos neonatais precoces ^12^ .

Lawn et al. ^15^ , em panorama mundial da mortalidade infantil no ano 2000, apontaram que ¾ dos óbitos neonatais ocorreram na primeira semana de vida. Nos 20 anos anteriores, ocorreu redução significativa da MIT e uma redução menos intensa, de apenas 25%, na mortalidade neonatal, com progresso pouco significativo na MINP. Ademais, para países com alto índice de desenvolvimento, a taxa de mortalidade neonatal por mil nascidos vivos era de quatro óbitos; na região das Américas, foi três vezes maior ^15^ .

Na análise da evitabilidade, chama atenção que 75% dos óbitos neonatais precoces foram atribuídos a causas evitáveis relacionadas à atenção à mulher na gestação, ao parto, e ao cuidado ao feto e ao recém-nascido. Esse primeiro dos três subgrupos de causas evitáveis foi responsável pela maior parte dos óbitos e apresentou evidente tendência de redução. Esse resultado pode ser decorrente da elevação do acesso à atenção pré-natal, pois ocorreu progressivo aumento da cobertura das equipes de saúde da família no estado entre 2008 e 2017: de 26,4% para 39,4% ^16^ .

Já o segundo subgrupo de óbitos, reduzíveis por adequada atenção à mulher no parto, não apresentou redução das taxas no período. Na atenção ao parto, destacou-se nesse intervalo a instituição de uma rede de atenção à saúde materna e infantil a partir de 2011 (Rede Cegonha) ^17^ ; entretanto, em avaliações de implantação, encontrou-se dificuldade para a sua efetivação e qualificação dos processos assistenciais em diferentes regiões do Brasil ^18^^–^^20^ .

O subgrupo de causas evitáveis reduzíveis por adequada atenção ao feto e recém-nascido apresentou significativa tendência de redução. Para esse subgrupo, seria importante uma análise posterior considerando o potencial impacto da Rede Cegonha ^17^ na expansão das ofertas de cuidados direcionados ao recém-nascido.

O grupo das demais causas (não claramente evitáveis), que participou com 22% a 24% do total dos óbitos neonatais precoces, não apresentou tendência significativa de redução das taxas no período estudado. Em estudo de abrangência nacional realizado por Malta et al. ^21^ no período entre 1977 e 2006 considerando a totalidade dos óbitos infantis, também não foi observada redução significativa nas taxas brutas, mas com redução significativa após ajuste pelas causas mal definidas.

Estudo analisando a mortalidade infantil no Brasil no período de 2000 a 2010 ^22^ aponta, na MINP, para a necessidade de reforçar a atenção à gestante, ao parto e ao recém-nascido. Entre os óbitos ocorridos no primeiro de vida, o baixo peso ao nascer e a prematuridade aparecem como os fatores de maior destaque. Embora parcela importante dos partos prematuros ainda não seja passível de prevenção, já é estabelecida a relação com tabagismo, infecção do trato urinário ou bacteriúria assintomática, passíveis de intervenção no atendimento pré-natal, como fatores para a redução da prematuridade. Outro ponto a ser considerado como fator para a prematuridade (e eventual óbito) é “a prematuridade iatrogênica devido à interrupção indevida da gravidez” ^23^ em um cenário em que as taxas de parto cesáreo têm se mantido elevadas ^4^ .

Na comparação entre as regiões do estado, fica evidente a heterogeneidade no comportamento das taxas e de suas tendências temporais. Em estudo acerca das causas evitáveis de morte neonatal no estado de São Paulo, Nascimento et al. ^23^ observaram heterogeneidade na distribuição dos óbitos neonatais nas diferentes regiões do estado de São Paulo; considerando as mortes por causas evitáveis, apontaram para algumas regiões que deveriam receber mais atenção.

Nos óbitos reduzíveis por adequada atenção à mulher na gestação, embora em 11 das 18 regiões estudadas tenha ocorrido tendência de redução na mortalidade, somente em duas regiões essas tendências apresentaram significado estatístico. Embora não existam dados sistematizados acerca da qualidade da atenção pré-natal oferecida nas diferentes regiões do estado de São Paulo, os dados de cobertura populacional da Estratégia Saúde da Família e da atenção básica como um todo, obtidos no site e-Gestor Atenção Básica ^16^ , mostram diferenças importantes na cobertura da estratégia, que é maior nas regiões a oeste e sul do estado, bem como nos municípios de menor população, decrescendo nos municípios maiores.

Vale lembrar que as equipes da Estratégia Saúde da Família são compostas nuclearmente por médico de saúde da família, enfermeiro, auxiliar ou técnico de enfermagem e agentes comunitários de saúde. Por outro lado, as demais equipes, ditas “tradicionais”, habitualmente possuem médicos nas especialidades de pediatra, ginecologia/obstetrícia e clínica médica, além de enfermeiro e auxiliar ou técnico de enfermagem. As diferentes composições e perfis profissionais existentes entre esses dois tipos de equipes podem produzir resultados distintos no atendimento às gestantes.

No entanto, o comportamento das taxas de mortalidade neonatal precoce evitáveis pela adequada atenção à mulher na gestação não guarda relação com a oferta (cobertura populacional) de atenção básica na modalidade de saúde da família. Estudo ecológico acerca da efetividade da estratégia em indicadores de saúde da criança no estado de São Paulo ^24^ não encontrou relação entre cobertura de saúde da família e taxas de mortalidade infantil neonatal; coberturas da acima de 50% evidenciaram efeito protetor apenas para o componente pós-neonatal da mortalidade infantil. Em relação aos óbitos neonatais precoces evitáveis por adequada atenção à mulher no parto, chama a atenção a evolução desfavorável no município de São Paulo, que soma cerca de 28% dos nascidos vivos no estado, embora a maior parte das regiões tenha obtido redução importante em suas taxas.

Um último ponto a ser considerado é o relativo à própria classificação de evitabilidade utilizada. Dias et al. ^25^ mostram resultados de análise de evitabilidade de óbitos infantis utilizando comparativamente diferentes métodos de classificação, chamando a atenção para a necessidade de atualização periódica destas classificações diante das permanentes mudanças tecnológicas na assistência.

O agrupamento das causas de óbito permitiu visualizar o impacto das ações dos serviços de saúde sobre a MINP. Ao se considerar apenas os óbitos antes do sétimo dia de vida, a participação de determinantes relacionados a acesso e qualidade das ações em saúde deve ser predominante, embora outros fatores possam também estar concorrendo para esses óbitos. Se a análise das tendências aponta para uma provável melhora no acesso e/ou qualidade da assistência à gestante e ao recém-nascido, a análise das causas de óbito agrupadas não permite visualizar possíveis pontos de intervenção nos serviços de saúde para a sustentação dessas tendências, bem como para o enfrentamento dos óbitos evitáveis por adequada atenção à mulher no parto, que não apresentou redução significativa no período.
